# Respecting living kidney donor autonomy: an argument for liberalising living kidney donor acceptance criteria

**DOI:** 10.1007/s40592-022-00166-4

**Published:** 2022-12-09

**Authors:** Alison C. Weightman, Simon Coghlan, Philip A. Clayton

**Affiliations:** 1 Adelaide Medical School, University of Adelaide, Adelaide, Australia; 2 Australia and New Zealand Dialysis and Transplant (ANZDATA) Registry, South Australian Health and Medical Research Institute (SAHMRI), Adelaide, SA 5000 Australia; 3 Central and Northern Adelaide Renal and Transplantation Service, Royal Adelaide Hospital, Adelaide, 5000 Australia; 4 Centre for AI and Digital Ethics, School of Computing and Information Systems, University of Melbourne, Melbourne, Australia

**Keywords:** Autonomy, Clinical decision making, Donor selection, Living kidney donation, Paternalism

## Abstract

Doctors routinely refuse donation offers from prospective living kidney donors with certain comorbidities such as diabetes or obesity out of concern for donor wellbeing. This refusal occurs despite the ongoing shortage of kidney transplants and the superior performance of living donor kidney transplants compared to those from deceased donors. In this paper, we argue that this paternalistic refusal by doctors is unjustified and that, within limits, there should be greater acceptance of such donations. We begin by describing possible weak and strong paternalistic justifications of current conservative donor acceptance guidelines and practices. We then justify our position by outlining the frequently under-recognised benefits and the routinely overestimated harms of such donation, before discussing the need to respect the autonomy of willing donors with certain comorbidities. Finally, we respond to a number of possible objections to our proposal for more liberal kidney donor acceptance criteria. We use the situation in Australia as our case study, but our argument is applicable to comparable situations around the world.

## Introduction

Kidney transplants produce substantial improvements in both quality and length of life for people with kidney failure (McDonald and Russ 2002, Tonelli et al. 2011). In Australia, most kidney transplants come from deceased donors; only 22% of kidney transplants are from living donors (ANZDATA Registry 2020). This is problematic for two key reasons. Firstly, in many cases, a kidney transplant from a living donor will be superior to an organ from a deceased donor because the former functions for longer, keeping those recipients healthier and off dialysis for a greater period of time (Mandal et al. 2003). Secondly, the number of patients with kidney failure awaiting transplants in Australia is expanding at a proportionally greater rate than can be met by current organ supply. Despite a progressive increase in deceased donor transplants in Australia, the last four years has seen a 30% increase in the number of patients on the waiting list for a deceased donor kidney transplant (ANZDATA Registry 2020). Meanwhile, living kidney donation rates have remained stagnant, representing an underutilised resource that could address this shortfall. Prima facie, these are compelling reasons for increasing rates of living kidney donation.

Much of the growth in deceased donor transplantation has occurred through liberalisation of deceased donor acceptance criteria, whereby kidneys from older and more medically complex donors who have died are accepted for use in transplantation. However, a similar liberalisation has not occurred for living donation, partly because of the unique ethical issues it raises. In particular, living donation creates an apparent conflict between a duty of beneficence (towards the transplant recipient) and a duty of non-maleficence (towards the living transplant donor). For this reason, and notwithstanding widespread acceptance of living donation, assessment of any living kidney donor remains an ethically fraught issue for many doctors, particularly when considering potential donors with pre-existing medical conditions (Spital 2001; Scheper-Hughes 2007; Wilkinson 2011).

Current practice requires that donors be in near perfect health to minimise their likelihood of experiencing harm following donation. This means that voluntary donors with pre-existing health problems, including diabetes, high blood pressure and obesity, will frequently have their decision to donate overridden by the transplant doctor responsible for assessing prospective donors prior to surgery. Altering current practice to accept more of these medically complex donors would enable more living donor transplants to take place. Yet many transplant doctors implicitly hold that the medical risk to donors of living kidney donation should always or at least typically override the wishes of living donors, especially for donors who have less than perfect health (which we call “non-ideal” donors).

In this paper, we argue that the current approach by transplant doctors to donor selection is too conservative and should, within limits, be liberalised. We argue that current approaches overemphasise the risks of kidney transplantation for the donor without giving sufficient weight to the benefits of donation for the recipient and, in many cases, the donor. In addition, current practice gives insufficient weight to the individual risk tolerance of the potential donor and thus fails to respect their autonomy. Liberalising donor acceptance criteria is therefore ethically justifiable and required on the grounds of proper appreciation of the widely accepted principles of beneficence, nonmaleficence, and respect for autonomy. In our view, the current exclusion of certain potential donors constitutes unacceptable paternalism: it overrides or gives insufficient weight to the informed choices of potential donors on the grounds that those people, by acting altruistically, could harm their own long-term health, even when all or many of these risks are low or unlikely to eventuate.^1^

In what follows, we first provide a brief overview of current living donor assessment practices and guidelines in order to highlight the ethical issues raised by excluding the majority of medically complex donors. We next outline reasons for paternalistic rejection and then justify the liberalisation of living donation criteria by appealing to the principles of beneficence, non-maleficence, and respect for autonomy, which are widely accepted in bioethics. Finally, we explore and refute several potential objections to our position. We use the situation in Australia as our case study. However, our argument is applicable to comparable situations around the world in which medically complex, living, potential donors are unjustifiably prevented from donating kidneys.

## Current legal and ethical requirements for living kidney donation in Australia

In Australia, the *Human Tissue Act* (1982) of each state and territory gives legal authority for an adult aged 18 years or older to consent to the removal of an organ for the purpose of transplantation, provided that a doctor has explained to the potential donor the implications of the removal and provided that the doctor is satisfied the person is of sound mind and is able to give their consent freely. Further to this, the Transplant Society of Australia and New Zealand (TSANZ) and the National Health and Medical Research Council (NHMRC) have developed guidelines that give more specific directives about living donor selection. These guidelines make it clear that the transplant doctor is to act as gatekeeper for access to living kidney donation and should uphold stringent parameters governing permissible donation (NHMRC 2007; TSANZ 2019). While deviation from these guidelines does not hold the same risks of prosecution and punishment as breaking criminal law, the guidelines nonetheless reflect and promote conventionally accepted practices by kidney transplant doctors in Australia. Furthermore, the guidelines set standards by which Medical Boards judge the professionalism of transplant doctors and can penalise and reprimand them for professional misconduct.

The *NHMRC Living Organ Donation Guidelines for Ethical Practice* state that living donation is only acceptable when “there are minimal risks of short and long-term harm to the donor, with no clinically significant loss of a bodily function.” (NHMRC 2007, p. 6). To adhere to this principle, prospective donors must meet rigorous selection criteria. As per Australian guidelines, kidney donors ought to have normal kidney function (defined as a GFR > 80 mL/min), normal blood pressure not requiring more than one medication, normal body weight (BMI < 30 kg/m^2^), and no history of diabetes mellitus or its precursors (Boudville and Isbel 2010; Cohney et al. 2010; Ierino et al. 2010; Isbel 2010). It is also the responsibility of the transplant team to ensure that the decisions of the donors are free from coercive influences including “undue emotional pressures” (NHMRC 2007, p. 6). According to the NHMRC guidelines, those people who articulate “emotional motives” for donation are required to be more carefully assessed; people who are “too willing” to donate are portrayed as raising special concerns, on the basis that emotional pressures can lead “well motivated people to take excessive risks” (NHMRC 2007, p. 28).

The strongly risk-averse nature of the NHMRC guidelines likely perpetuates moral uneasiness among transplant doctors about accepting any living kidney donors, but especially donors who have less than perfect health. Studies from Australia and overseas suggest that 41–68% of potential donors do not proceed to donate following evaluation, usually due to exclusion by transplant doctors on medical grounds (Riehle et al. 1990; Mağden et al. 2015; AlBugami et al. 2019; Cash et al. 2019; Altheaby et al. 2020). Given these high rates of donor rejection, it is worth considering whether these guidelines are excessively conservative and whether doctors, by trying to adhere to such restrictive criteria, are unacceptably violating the autonomy of willing donors and depriving potential recipients of a major medical benefit. Indeed, some overweight and pre-diabetic donor candidates, for whom donation is deemed to be contraindicated in Australia, would be permitted to donate in other comparable medical systems, such as in the United Kingdom and the United States of America (Tong et al. 2011; Thiessen et al. 2015).

It is also important to consider the professional ethical obligations of the transplant doctors, including their responsibility to “protect and promote the health of individuals,” as the Medical Board of Australia’s professional guidelines state (Medical Board of Australia 2020). This directive to doctors is underpinned by the long-recognised ethical principles of beneficence and non-maleficence (Miles 2005). However, it is widely accepted that these principles are not absolute and that their application is affected by relevant circumstances and by other ethical principles (Beauchamp and Childress 2019). Accordingly, in some circumstances the usual requirement for doctors to prevent harmful outcomes for patients is overridden by the requirement to respect patient autonomy.

When assessing potential living donor candidates, transplant doctors are professionally expected to promote the wellbeing of both donor and recipient as well as to protect both parties from harmful outcomes. Doctors are also obligated to respect the autonomy of recipients and donors, such as by allowing a living donor who initially consents to donation to withdraw from the program should they change their minds. At times, the obligation to protect patients from harm can prompt doctors to override an individual’s autonomous decision to donate. While this constitutes paternalistic action, this paternalism is not necessarily wrong (Goldfarb 2019). For example, most people would agree that the permissibility of a doctor’s actions in overriding a donor’s wishes to altruistically give up an organ is rightly affected by the magnitude of harm the donor faces. If donation was likely to result in the donor’s death, for instance, paternalistic refusal would be justified, even if this would mean that a potential recipient does not receive a vital health benefit. However, many donors are faced with less extreme and/or less certain risks of harm. For example, it is recognised that some donors will develop protein in their urine as a consequence of donating a kidney, an asymptomatic condition with unclear implications for the future kidney health and overall wellbeing of those donors (Hansen 2019). Given the uncertainty about outcomes for these donors, the guidelines recommend exclusion of these donors in favour of extremely low risk donors who are unlikely to develop protein in their urine. It is these sorts of examples of paternalistic donor exclusion which we seek to challenge.

To assess whether the current level of paternalism in countries like Australia is justified, we need to understand the possible and likely benefits and harms of kidney donation from individuals with mild to moderate conditions and how to balance them. We also need to understand the relevance of the principle of respect for autonomy to this case.

## Ethical reasons for conservatism in donor selection

As outlined above, the current Australian transplant guidelines broadly support a paternalistic approach toward potential living kidney donors, by placing the onus on the transplant doctor to determine the permissibility of donation offers based on the doctor’s interpretation of the patient’s best interest and by not recognising the possibility of accepting donations from individuals with certain mild to moderate comorbidities. Transplant doctors can decline donation offers from healthy individuals if the doctor believes there is an increased risk of the donor developing kidney disease or other chronic illnesses later in life, regardless of the objective magnitude of these risks or of whether the potential donor accepts those risks. The decision to proceed with a living kidney transplant is therefore conditional on the assessing doctor’s risk tolerance, rather than that of the potential donor.

Paternalism describes situations where Person A (the paternalist) intentionally substitutes their own decision-making regarding the interests of Person B, overriding or disregarding the wishes or desires of Person B, with the intent of benefiting or protecting the interests of Person B (Dworkin 2020). In living kidney donation, this could occur when willing donors are declined because the assessing doctor believes this decision to be against their interests. There are two important kinds of paternalism to consider here. *Weak* paternalism occurs when the doctor overrides the wishes of a patient who for various reasons cannot or does not give their fully autonomous (informed, voluntary) consent. *Strong* paternalism occurs with the overriding of fully autonomous decisions. Both weak and strong paternalism may be offered as defences of the current conservative donor criteria.

### Weak paternalism

There are several situations in which a doctor could justifiably claim that they had declined a donor because the donor’s request to donate did not reflect a truly autonomous decision. As the NHMRC guidelines say, doctors are required to critically examine all donor offers for voluntariness and to intervene in situations where the potential donor is being coerced into offering their organ (NHMRC 2007). For example, doctors should reject donor offers that have been elicited through threats of violence or manipulation. In this instance, refusal of a donor on the grounds of weak paternalism is perfectly appropriate. Doctors might also conceivably claim that potential donors were not acting autonomously because they were excessively influenced by emotion and were therefore unable to rationally weigh up the benefits and risks of kidney donation. This possibility is alluded to in the NHMRC guidelines when it cautions doctors to be wary of donors who, under the influence of emotional motivators, are “too willing” to take excessive risks (NHMRC 2007).

Accordingly, a doctor might claim that a person with pre-existing health conditions such as diabetes or obesity who wanted to donate a kidney in fact did not correctly understand the implications of that decision. Indeed, some doctors may believe that certain donors with mild or moderate comorbidities (e.g. obesity, controlled diabetes) are in no position to make *autonomous* decisions about donation, because those donors are mostly or always suffering from misunderstanding or irrationality in choosing to donate despite their higher risk status. Transplant doctors have frequently expressed concerns about patients’ abilities to interpret complicated medical information and statistics correctly when making decisions about kidney transplants (Cardinal et al. 2020; Tong et al. 2013). Furthermore, several landmark studies have demonstrated that presenting scientific evidence to potential donors regarding the risks of donation did not alter the vast majority of potential donors’ decisions to donate, raising concerns about the apparent lack of impact of medical risk on donor decision making (Fellner and Marshall 1968; Simmons et al. 1977; Lennerling et al. 2003). Doctors might therefore argue that non-ideal donors were not making an autonomous decision: had these riskier donors correctly understood the implications of donating their kidney, they would not have chosen to proceed.

However, this justification of non-ideal donor exclusion on the basis of weak paternalism is difficult to sustain as there is no evidence or reason to suspect that non-ideal donors are less able than ideal donors to make autonomous decisions about donation. Non-ideal donors are just as likely to be driven by emotional motives and are subject to the same risk perception limitations as ideal donors. Furthermore, it is generally accepted that valid consent can be obtained even in the absence of perfect comprehension of risks (Beauchamp and Childress 2019; Faden and Beauchamp 1968). For example, a prospective donor might accept the risks of surgical complications and kidney dysfunction without being able to grasp the precise nature and magnitude of those risks, and yet still be acting autonomously. This is just as true of donors with comorbidities as it is of donors without them. Interestingly, interviews with transplant doctors indicate that doctors are sympathetic to the emotional motivators that inspire non-ideal donors to seek donation and are more likely to bend the rules for them (Tong et al. 2013). For example, doctors report they are more likely to accept a non-ideal donor in the setting of a spousal or parental donation scenario than a person wanting to donate to a friend or stranger (Tong et al. 2013). This shows that doctors sometimes accept that donors with co-morbidities are acting autonomously. In sum, insofar as it partly underpins current practice, weak paternalism is a poor defence of donor guideline conservatism.

### Strong paternalism

A second ethical reason for the current conservatism is so-called strong paternalism, whereby doctors choose to overrule the decisions of autonomous non-ideal donors for their own good. Indeed, when interviewed, transplant doctors articulated strong paternalist rationales for excluding non-ideal donors, expressing a sense of responsibility for safeguarding long-term donor health (Tong et al. 2013).

Strong paternalism is certainly a more promising defence of conservatism in living kidney donation than weak paternalism. Beauchamp and Childress provide a consequentialist defence of some instances of strong medical paternalism. They advance three criteria for assessing whether a paternalistic action could be acceptable: (1) when there is a serious and preventable risk of harm to the patient that would likely be prevented by the paternalistic action; (2) when the expected benefits of the paternalistic action outweigh the harms; and (3) when the action taken is the least autonomy restricting alternative (Beauchamp and Childress 2019).

The key question we address in the remainder of this essay is whether strong paternalism can justify current conservative practice. We can deal with Beauchamp and Childress’s third criterion about minimising the degree of autonomy restriction briefly. Preventing donors from donating altogether is hardly an action that admits degrees in the restrictions it places on autonomy. Nonetheless, partially autonomy-preserving alternatives could perhaps include opportunities for donors to appeal unfavourable decisions. Arguably, potential donors might also be presented with less restrictive methods than flat rejection of discouraging allegedly risky donations, such as through education or discussions with patient peers. However, these approaches still restrict the exercise of autonomy in significant ways, by rejecting wishes for donation altogether or by nudging would-be donors in other directions. Current approaches significantly reduce opportunities for compromise in risk tolerance between the potential donor and the transplant team.

Beauchamp and Childress’s first justifying criterion of preventing a serious risk of harm—in this case harm to donors with comorbidities—requires us to consider both the likelihood and magnitude of the risks posed towards a particular donor. Beauchamp and Childress’s second criterion of justified strong paternalism refers to the harms and benefits of paternalistic actions.

Now, a﻿ defender of the status quo could say that the likelihood and magnitude of risks to the non-ideal donor are too great to outweigh the possible benefits of that practice, and that such paternalism does not result in excessive harms.

We should acknowledge that the donor is asking the doctor to cause them known and sometimes unknown risks of harm, including a very small chance of serious or catastrophic harm. Furthermore, unlike most doctor-patient interactions, these risks are borne by the donor without the compensation of receiving any of the medical benefits that normally justify medical interventions. Indeed, while the benefits of living kidney donation for recipients are often significant, the benefits for the donor are apparently either non-existent or very modest. The strong paternalist about kidney donation could therefore argue that even a small increase in risks, as might be experienced by a non-ideal donor, could be sufficient to tip this balance in favour of paternalistic exclusion of such donors. To respond to these arguments for strong paternalism, we turn to a more detailed analysis of the benefits and harms of donation in relation to non-ideal donors. Subsequently, we take up the question of respect for donor autonomy.

### Benefits for recipient

The benefits of living kidney donation for transplant recipients are significant. Recipients of living donor transplants live longer than recipients of deceased donor transplants, with 67% still alive at 20 years compared with 45% of deceased donor recipients (ANZDATA Registry 2020). Furthermore, kidney transplants from living donors remain functional for longer than those from deceased donors—lasting on average 20 years in comparison to 15 years for deceased donor recipients (ANZDATA Registry 2020). This leads to greater gains in general health from not being on dialysis as well as major improvements in quality of life (Purnell et al. 2013). There are also fewer transplant complications (e.g. rejection) due to the planned nature of living donor surgeries, better immune matching, and avoidance of the physiological trauma to the transplant that is associated with donor death (Reese et al. 2015).

Living donor transplant recipients are also able to access transplantation earlier, with many transplants occurring before the recipient needs to start dialysis (Milton et al. 2008). While dialysis is a life sustaining treatment, it contributes to many health complications including heart and bone disease. Patients who spend no time or less time on dialysis accrue fewer of these related illnesses. Additionally, patients who receive a living donor kidney can avoid the deceased donor waiting list and will therefore usually get a transplant much earlier. This correlates with better recipient outcomes by minimising time spent on dialysis. For some dialysis patients, living donation can represent the only realistic option to access a kidney transplant. This includes older patients who are unlikely to survive a protracted period on the deceased donor waiting list (Gill et al. 2008).

By permitting a greater number of non-ideal donors, rates of living kidney donation could be increased, enabling a greater number of recipients to receive a superior transplant organ while spending less time on dialysis. Additionally, living kidney donation reduces demand for deceased donor kidney transplants, as recipients with a living donor do not require access to this scarce organ. The benefit of this would be decreased time on the deceased donor transplant waiting list for recipients who do not have a suitable living donor option. In sum, the substantial benefits for recipients (and potentially other parties) present a compelling prima facie argument for more liberal donor selection.

### Harms and benefits for donor

In sharp contrast to the transplant recipient, it may appear that the willing kidney donor stands only to be harmed from their altruistic donation of an important organ. Donors undergoing surgery typically experience pain and limitation of movement during recovery for up to 6–8 weeks following the surgery (Nicholson et al. 2010). They are often unable to work for several months, which can cause financial losses (Fu et al. 2020). A proportion of donors will experience more serious complications including infections, delayed wound healing or accidental damage to other organs such as spleen or bowel, which can result in longer recovery times and reduced quality of life (Wilson et al. 2011; Lentine et al. 2019). Additionally, all donors face some risk of catastrophic harms such as kidney failure or death due to surgical or anaesthetic complications (Segev et al. 2010). Large retrospective studies have shown that the absolute risk of major harm such as kidney failure or death is very low, at 0.9% and 0.03% respectively (Segev et al. 2010; Muzaale et al. 2014; Lentine et al. 2019).

When turning down donation offers from non-ideal donors, doctors are often motivated by non-maleficence based concerns that these individuals are at higher risk of future kidney failure (Tong et al. 2013). Clearly, a person who donated *both* kidneys would face the certainty of catastrophic kidney failure. However, an individual with a health condition that could predispose them to kidney disease has a much lower risk of suffering a very bad outcome (Steiner 2004). While data in non-ideal donors is limited by their exclusion from routine donation, several smaller studies of non-ideal donors with less severe co-morbidities such as obesity, impaired glucose tolerance or hypertension show similar rates of major complications like kidney failure or death as compared with ideal donors (Kumar et al. 2003; Goldfarb 2005; O’Brien et al. 2012a, b; Okamoto et al. 2010).

Uneasiness around permitting non-ideal donors relates in part to uncertainties about their short- and long-term prognoses, including the risk of major and minor harms. In particular, there is a lack of evidence about long term outcomes as a result of their long-standing exclusion as donors (Ahmadi et al. 2015). For example, recommendations for exclusion of diabetic donors are derived from observations in animal models; the actual risks to diabetic donors are as yet unquantified, even in non-transplant patient cohorts (e.g. patients undergoing nephrectomy for renal cancers) (Chapman et al. 2010). Doctors express concern about extrapolation of existing study information to potential donors with co-morbidities for fear it could underplay the magnitude of the harms they are facing (Tong et al. 2013). However, there is also some acknowledgement that strict exclusion of donors with unknown risk profiles is potentially excessive and unjustified as the risk of harm may be overstated (Steiner 2004; Tong et al. 2013).

The key point we make here is that there is evidence and reason to believe that the *additional* medical risks for donors with certain mild or moderate morbidities compared to ideal donors are relatively small. Most doctors and ethicists already think that kidney donation from ideal donors is morally justified despite the medical risks they face. Given that they are correct in thinking this, the next question is whether the small additional risks for non-ideal donors are morally outweighed by other ethical considerations. We believe that they are indeed outweighed and that this requires liberalisation of donor selection.

To this end, it is also important to note that the risk-oriented perception of impact on donors represents a one-dimensional understanding of health that is at odds with more holistic perspectives. In fact, when we adopt a wider, liberal notion of benefit than strict medical benefit, it is evident that donors very commonly benefit from their act of donation. Many donors, for example, experience an improvement in reported wellbeing after donation. Indeed, more than 90% of donors reported improved quality of life and self-esteem following the donation and had high levels of satisfaction with their decision to donate (Smith et al. 1986; Schover et al. 1997; Hartmann et al. 2003). These outcomes translate into enduring psychological benefits for the vast majority of donors, and contributes to a greater quality of life.

Perhaps surprisingly, this positive effect is true even of donors who experience certain major complications. It has been shown that when donors experience negative outcomes (e.g. kidney failure), many do not regret their decision to donate and would make the same choice again (Hartmann et al. 2003). Furthermore, refusal to allow a donation to take place can cause harm to donors. Declined donors report feeling disappointed and ashamed, with 32% of declined donors reporting their life was worse after discovering they were not able to donate (Agerskov et al. 2015; Hanson et al. 2017; Reese et al. 2018).

Donors can also derive benefits from the improved health of the recipient, as most donors are related or married to their recipient. Many family members report social and financial stressors from having a relative on dialysis (Hoang et al. 2018). Relatives must also sometimes contend with the persistent awareness of the ill health of the patient on dialysis and the limitations this places on their own ability to work and travel (Tong et al. 2012). This is frequently cited by donors as a motivator to come forward for assessment for living kidney donation (Lennerling et al. 2004; Tong et al. 2012). Successful transplantation can significantly reduce this burden of care placed on families.

For the above reasons, we should be careful not to overestimate the harms caused to living donors by their donation. First, the harms and risks are relatively small; and second, most donors benefit overall from their actions. Nonetheless, kidney donation from donors with co-morbidities does involve some non-trivial degree and risk of harm to the donor. Indeed, we believe that justifying this practice requires going beyond this balancing of harms and benefits. More specifically, we contend that a proper respect for autonomy in this context is not only a necessary condition of accepting donations from autonomous persons, it also (along with the provision of significant benefits to the recipient) helps to outweigh the prima facie duty of nonmaleficence towards potential donors.

## Respect for autonomy

Australian law requires living organ donors to be competent adults over the age of 18 years. This means that individuals wishing to be donors must have the capacity to express their autonomous wishes regarding the procedure. Respect for the donor’s autonomy entails that transplant doctors give weight to their considered views when weighing up whether to proceed to kidney donation. However, respect for autonomy in the guidelines for living donor transplantation is construed purely as a negative right, to be exercised when preventing donations from people who do not consent. For example, when discussing autonomy, the NHMRC ethical guidelines state that the living donor’s autonomy must be given precedence over the recipient’s need to receive an organ, to ensure protection of the donor’s right to refuse donation at any time before the operation (NHMRC 2007). Yet there is no discussion in the guidelines about respecting positive rights, such as by acceding to or even promoting the autonomous wishes of donors who wish to donate in the face of some relative contraindications such as diabetes and obesity.

Modern relational views of autonomy in medicine highlight the role of the doctor-patient partnership in decision-making (Ross and Thistlethwaite 2018). On these views, the doctor is not just a source of information for patients, nor merely a guardian against harmful decision-making; instead, the doctor has a role in actively assisting the patient to make choices that align with their values. For the prospective living kidney donor, this conception of autonomy requires the transplant doctor to partner with the potential donor in jointly exploring individualised risks and benefits and to support choices that align with the donor’s principles, such as those concerning their tolerance for personal risk in the light of their various values and preferences.

This is particularly relevant when considering potential donors with pre-existing medical conditions that, on current standards at least, contraindicate donation. A competent donor who is for instance significantly overweight or diabetic may believe that their risk from donation is sufficiently outweighed by the anticipated benefits to themselves and/or the recipient of kidney donation. Assuming such a decision aligned with their values, the principle of respect for autonomy would presumably lend strong support to allowing such a person to proceed with donation.

It is true that if the risk of harm to the donor was sufficiently high, then duties of nonmaleficence may outweigh respect for autonomy. This is one reason why we would oppose the indiscriminate acceptance of all potential donors. There will still be some autonomous potential donors for whom the loss of one kidney poses unacceptably high risks of significant and permanent harm or death, and in such cases strong paternalism is justified. Yet when the risks and harms to potential donors are not so severe and are in fact mild or moderate, or indeed are even outweighed by the other benefits they typically receive, respect for donors’ autonomy (in combination with the likelihood of significant benefits to recipients) justifies liberalising current practice. This could be the case, for example, for donors currently excluded based on obesity, hypertension, or milder forms of diabetes and proteinuria.

It is important to stress that we (doctors, ethicists, etc.) overwhelmingly already accept a degree of risk in *ideal* donors. Given that the additional risks to many non-ideal donors are relatively small and often accompanied by significant benefits for them, and given the role in this context of the principles of beneficence and respect for autonomy, we believe the case for rejecting the current level of paternalism in transplant practice is convincing.

## Summary of the case against current paternalism

The current Australian living donor guidelines encourage transplant doctors to act paternalistically, by excluding willing non-ideal donors on the grounds that kidney donation is not in their best interest. But there is no reason to believe that non-ideal donors are less able to have and articulate their autonomous wishes than donors in perfect health. Therefore, weak paternalism is an implausible defence of the status quo and should be rejected.

Strong paternalism offers a more plausible justification of present conservativism. Yet for strong paternalism to be justified, respect for potential donors’ autonomy and the duty of beneficence to recipients must be outweighed by the degree of risks and harms posed to the donor. We have presented evidence that this harm is overstated for many non-ideal donors, especially those with single and/or mild comorbidities. Furthermore, we argued that the benefits of donation are underrated due to a traditional non-holistic view of donor outcome that includes emotional, social, and economic benefits. We also claimed that the magnitude of the harm to be prevented does not outweigh the benefits to recipients to a degree that would permit the overruling of the non-ideal donor’s autonomy.

Finally, if ‘non-ideal’ donation were to be rejected on the grounds of nonmaleficence to the potential donor, then it would seem to follow that there is also reason to reject ‘ideal’ donation. But transplant doctors and relevant bodies (like the NHMRC) are not arguing for ending the practice of living donation. Thus, we conclude that paternalistic actions based on Beauchamp and Childress’s criteria for justified strong paternalism cannot be sustained.

## Objections to our position

Having presented our ethical arguments for liberalising current donation practice, we must now briefly consider potential objections against this proposed change for donors with certain comorbidities.

Potential opponents to our proposal could argue that allowing more donations from individuals with co-morbidities will result in greater numbers of donors experiencing adverse outcomes post donation. This, it could be argued, would undermine current statements regarding the safety of kidney donation and could damage public trust in the living donor transplant program, potentially resulting in the loss of future ideal donors.

We offer several replies to this argument. Firstly, we are not advocating for universal acceptance of all potential donors, and therefore we anticipate that most donors facing likely significant complications will be excluded. Hence, the overall rise in serious adverse outcomes such as kidney failure will be low. Secondly, informed consent requires that the transplant doctor provide personalised information regarding the individual risk profile of each donor. If doctors perform more non-ideal donations, we will be able to get more data regarding outcomes in non-ideal donors and hence be able to provide better risk forecasting for potential donors. This should aid in offsetting discontent regarding donor outcomes in the event that a negative health consequence occurs. Finally, most donations occur between close relatives (e.g. parents, spouses, siblings), with evidence suggesting that magnitude of risk does not affect the decision to donate (ANZDATA Registry 2020; Lennerling et al. 2003). It is therefore unlikely that a small increase in adverse outcomes will deter the majority of living kidney donations from taking place.

It could also be argued that expansion of living kidney donation exposes more people to the harms of nephrectomy and that this is unconscionable when there are other viable options for the treatment of renal failure, such as dialysis or deceased donor transplantation. This objection is also surmountable. Firstly, as outlined earlier, recipient outcomes from living kidney donation are superior to those who remained on dialysis or received transplants from deceased donors. In our view, this suggests that living donation should be promoted as the best option for kidney failure patients and should be supported by guidelines which encourage rather than impede willing volunteers becoming donors. It is worth noting that active promotion of living kidney donation over deceased donor transplants has been already adopted by other countries such as the United States of America and New Zealand (Waterman et al. 2015; Martin 2014). Furthermore, it is difficult to justify opposing non-ideal donors on these grounds without extending the opposition to all possible donors: if we are to believe that nephrectomy is an unacceptable harm, then we should believe this for both ideal and non-ideal donors. As we have stressed, the risks of harms for many non-ideal donors are only marginally greater than for ideal donors, according to the best current medical knowledge.

A doctor could argue that being pressured by more liberal guidelines to proceed with a donation against their professional judgment fails to adequately respect their own autonomy as the transplanting doctor. Indeed, doctors are permitted to refuse to participate in medical care that is at odds with their personal values (e.g. abortion or euthanasia) on the grounds of conscientious objection. On this view, transplant doctors are not mere instruments to facilitate transfer of one person’s kidney to another person; they are moral agents in their own right and should not ordinarily be compelled to act against their consciences.^2^ However, recognising the right to conscientious objection does not equate to preventing willing doctors from accepting donations from donors with certain comorbidities. A doctor who objects to allowing certain donors based on their personal moral views can, in line with current professional codes of practice regarding conscientious objection, refer such a patient to another transplant doctor for an independent opinion (Medical Board of Australia 2020).

Finally, there is considerable and understandable concern among organ transplant authorities about the possibility of there being living donors who involuntarily consent to surgery due to familial or social pressure or manipulation (Papachristou et al. 2011; Gordon 2012). Doctors are very aware of their limitations in detecting such occurrences and have voiced particular concern about undetected coercion in non-ideal donors where the operative and long-term health risks are potentially higher (Tong et al. 2013). This is not to say that non-ideal donors are believed to be more vulnerable to coercion than standard donors; rather, it is to acknowledge that when the stakes are higher, doctors tend to be more concerned about ensuring the decision to proceed with a higher risk donation reflects the true, uninfluenced wishes of the donor (Tong et al. 2013).

Certainly, when potential donors are subject to coercion or manipulation, their offers to donate should, all things being equal, be rejected, pending the possibility of restoring autonomous choice.^3^ However, this should be applied equally to both standard and non-ideal donors: non-ideal donors should not be subject to higher levels of scrutiny and scepticism regarding the presence of coercion. Doctors must be alert to not preventing non-ideal donations based on intuitions without sufficient evidence of involuntary consent, particularly if they would not have opposed the offer of a similar donor without co-morbidities. We therefore support the practice of providing non-ideal donors with the same safeguards from coercion that are offered to standard donors—for example, appointments that are conducted independently of the recipient and that allow ample and clear opportunities for the donor to withdraw their initial consent.

It is also worth noting that concerns about coercion of non-ideal donors tend to be unilaterally applied: there is relatively less concern about doctors paternalistically coercing potential donors into *declining* to donate out of concern for their welfare. But in the light of our arguments, if one is to oppose coercion of donors on the grounds of interference with autonomy, we one should also require that doctors refrain from applying pressure to dissuade willing non-ideal donors, even if the doctor believes that donation is not in that individual’s best interest. If those doctors cannot for personal moral reasons accede to such donation offers, they may respectfully exercise their right of conscientious objection and refer to another doctor.

## Conclusion

The superior performance of living donor kidney transplants and the worsening shortage of deceased donor transplants make a compelling case for increasing living kidney donation in Australia and comparable places by facilitating donations from appropriately informed and consenting donors with certain medical comorbidities. In this article, we have argued that such an approach is ethically justifiable on the grounds of a careful consideration of the principles of non-maleficence, respect for donor autonomy, and beneficence (including a liberal interpretation of donor benefit). We demonstrated that decisions by transplant doctors to reject autonomously consenting, medically complex donors can constitute unjustifiable paternalism. Therefore, current guidelines and practice for living kidney donation should be changed.

While we acknowledge that there are some people who face unacceptable harms from donation and therefore should not be permitted to donate even when they wish to, we reject the view that these possibilities are sufficient to justify ongoing exclusion of autonomous donors with certain mild to moderate comorbidities. Adopting this donor-centric approach to decision making for living kidney donation will facilitate an ethically justified increase in living kidney donation, to the benefit of the many kidney recipients and very often to the kidney donors themselves.

## Footnotes

1 It should be noted that this argument could also be used in support of potential donors who wished to autonomously sell their kidneys. We acknowledge that there is ongoing debate around the ethical permissibility of the commodification of organs, particularly in relation to the tension between paternalistic restrictions on kidney sales and the potential for harms from donor exploitation if such constraints did not exist. However, extending this justification to organ sales raises other complex issues, such as moving from a practice that views organ donation as a gift to one that sees organs at least in part as marketable commodities. We have limited our discussion to assessment and acceptance of living kidney donors within the existing system of altruistic donation as we believe this is a more immediate step to allow more living kidney donations to take place. See also: Koplin (2017) and Martin and White (2015).

2 Note, however, that some bioethicists would dispute this, e.g. Schuklenk and Smalling (2017). We cannot address this complex debate here.

3 We note in passing that the ethics of declining requests made under coercion are actually rather complex - for an in-depth analysis, see: Millum, J., (2014). Consent Under Pressure: The Puzzle of Third Party Coercion. *Ethical Theory and Moral Practice*, *17*(1), 113–12.
